# 

**Published:** 1814-04

**Authors:** 


THE
### Medical and Physical Journal.

4 OF VOL. XXXI.] APRIL, 1814. [NO. 182.

### The PROPRIETORS to the PUBLIC.

*IT has been the fate of the Proprietors of this original Journal, in common with the projectors of every successful undertaking, to witness the rise, decline, and fall of many rival works on a similar plan. They do not complain either of circumstances which are not peculiar to themselves, nor of the honorable and well-meant endeavours of others; always confiding for protection, against piratical copyists or insidious oppositions, in the just feelings of that intelligent part of the community to whom this Journal is addressed. They consider it due, however, not less to the* FACULTY *at large than to many old and valued Friends, and their own interests in particular, to assert the superior and undiminished pretensions of a work of established reputation and universal circulation, as a means of drawing together original Communications from all parts of the world, and of giving them that degree of currency which affords at once a maximum of gratification to the writers, and of utility to the profession and the public. On this point they venture also to urge the evident* CONVENIENCY *and* ECONOMY *of uniting all current medical improvements and correspondence in one periodical work; and they presume to urge the legitimacy of their own claims, founded on their* LONG ESTABLISHMENT, *their* PAST SERVICES, *their well-known* IMPARTIALITY *in whatever regards the interests and feelings of various branches of the profession, and their determination to concentrate within their pages* EVERY ORIGINAL FACT *and* VALUABLE OBSERVATION, *let them appear in whatever form or quarter they may.*

*They are duly sensible at the same time that the foundation of all their pretensions to public favor and preference is in the undeviating merit of their work; and they have, therefore, among other new arrangements, associated with* DR. FOTHERGILL *a colleague selected from another branch of the profession, and whose title to the approbation of their readers will, they trust, be made evident by the increased interest of their pages.*

### MONTHLY CATALOGUE OF MEDICAL BOOKS.

AIKIN'S (A. and C.) Account of the most important Discoveries and Improvements in Chemistry and Mineralogy to the present time, being an Appendix to their Dictionary of Chemistry and Mineralogy, with plates, 4to. 18s.

Clarke's (C. M.) Observations on those Diseases of Females which are attended by Discharges, illustrated by copper-plates of the Diseases, &c. Part 1, Mucous Discharges. Royal 8vo. 1l. 1s.

Singer's Elements of Electricity and Electro-Chemistry, with plates, 8vo. 16s.

Smith's Treatise on Glanders, being chiefly a plain Narrative of Facts, elucidating the Cause, Preventives, and Cure of that destructive Malady. 8vo. 7s. 6d.

Ware's Remarks on the Ophthalmy, Psorophthalmy, and Purulent Eyes, of New-born Children. Fifth edition, with many additions. To which is now added, an Appendix on the Purulent Ophthalmy, which has lately been epidemical in this country. Second edition. 8vo. 10s. 6d.

The Medical Guide for Tropical Climates, particularly the British Settlements in the East and West Indies, and the Coast of Africa. By Richard Reece, M.D. 8vo. 9s. boards.

A Treatise on Hernia. By Antonio Scarpa, Professor of Clinical Surgery in the University of Pavia. Translated from the Italian, with Notes and an Appendix. By John Henry Wishart, Surgeon. 8vo. 16s. boards.

A Treatise on the Hydrencephalus, or Dropsy of the Brain. By James Carmichael Smyth, M.D. 8vo.

